# Supplementary material for: Immune Pathways with Aging Characteristics Improve Immunotherapy Benefits and Drug Prediction in Human Cancer
Source: Cancers (Basel). 2023 Jan 4;15(2):342. doi: 10.3390/cancers15020342 (PMC9856581; doi:10.3390/cancers15020342)
Supplement: Supplementary file 1 [file cancers-15-00342-s001.zip › Supplementary Figures.pdf]

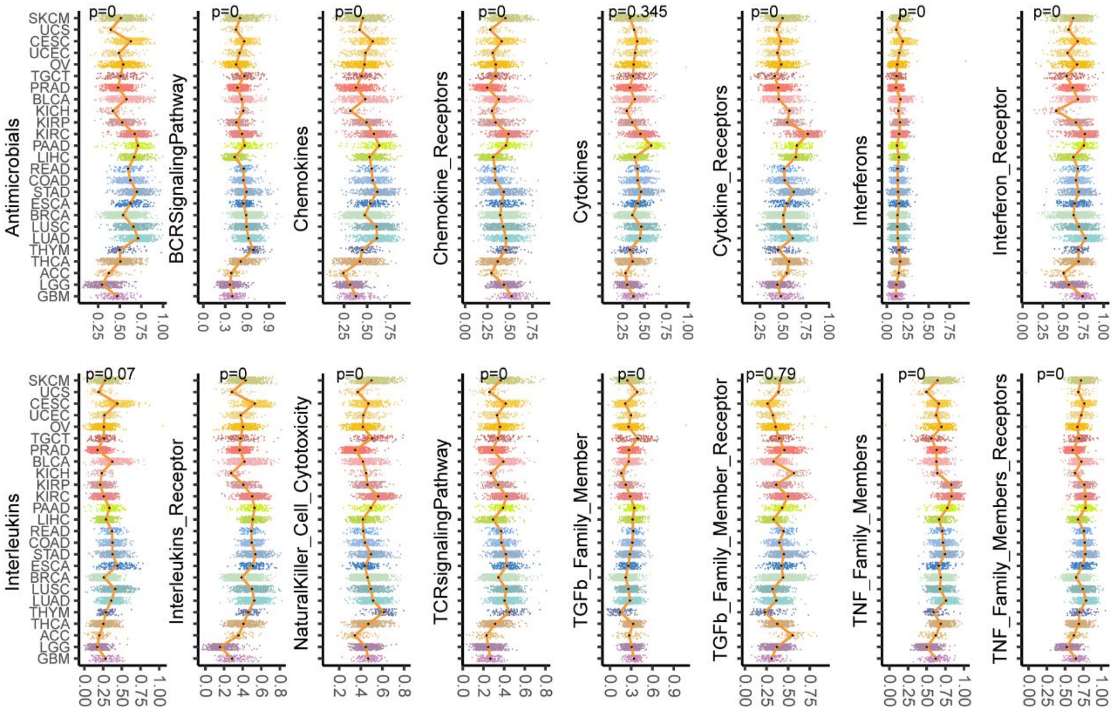

Supplementary Figure S1. Normalized IRP scores across 25 cancer types.

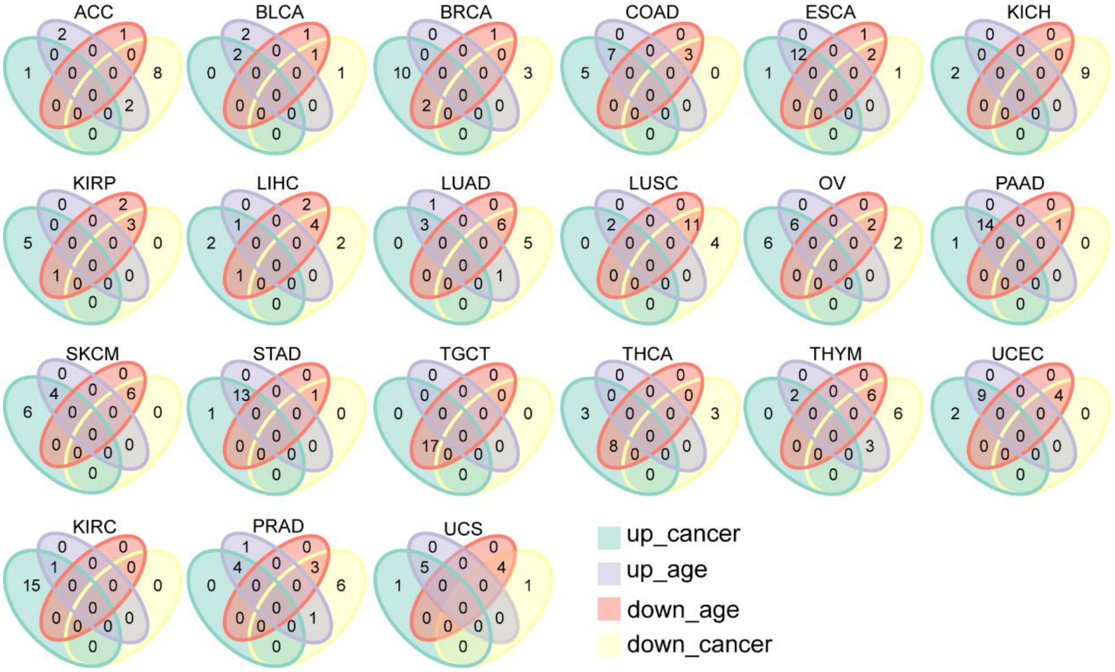

Supplementary Figure S2. Venn diagrams showing the overlap between dysregulated tumor- and age- IRPs.

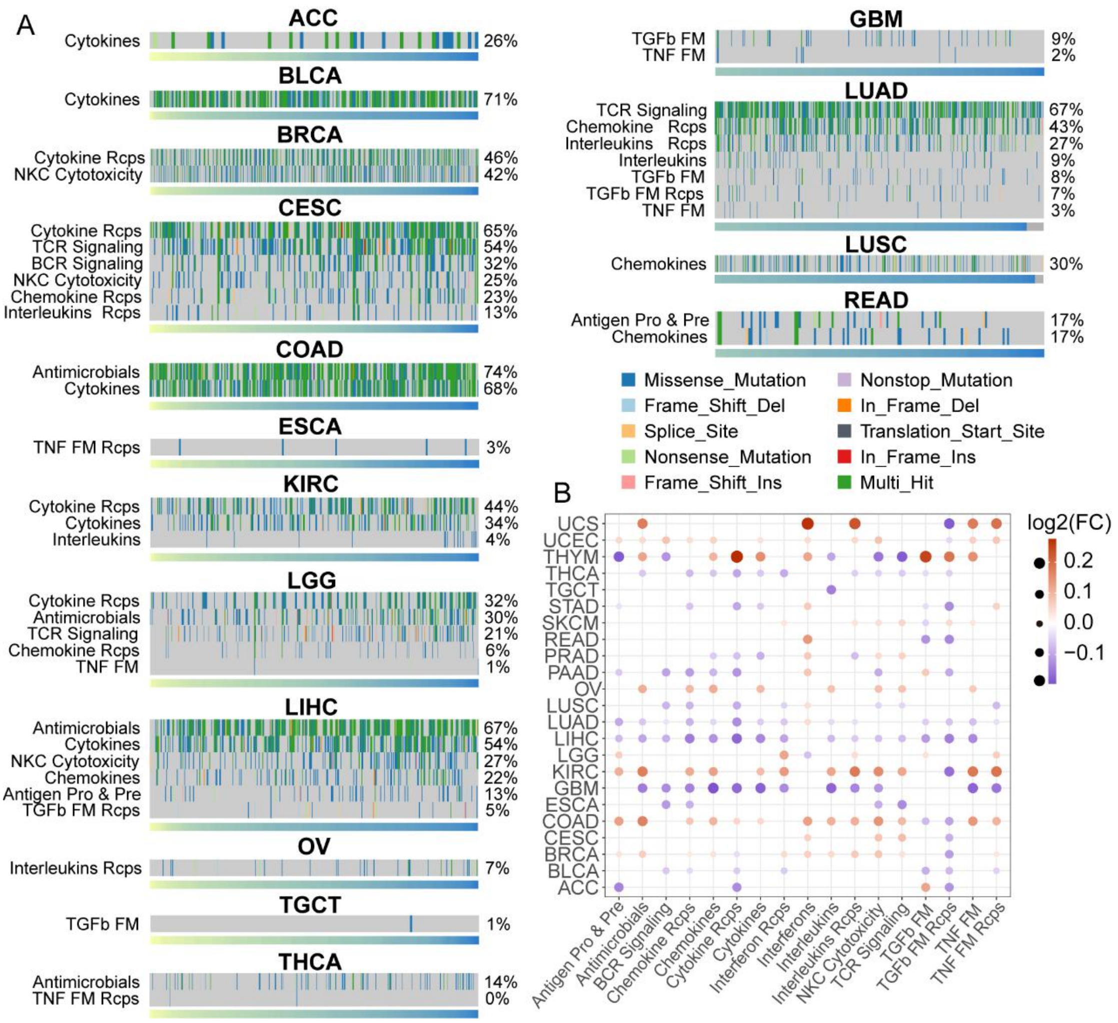

Supplementary Figure S3. A Oncoplot of IRPs in different cancers. **B** Dotplot of associations between IRP scores and mutations in IRPs across various cancers.

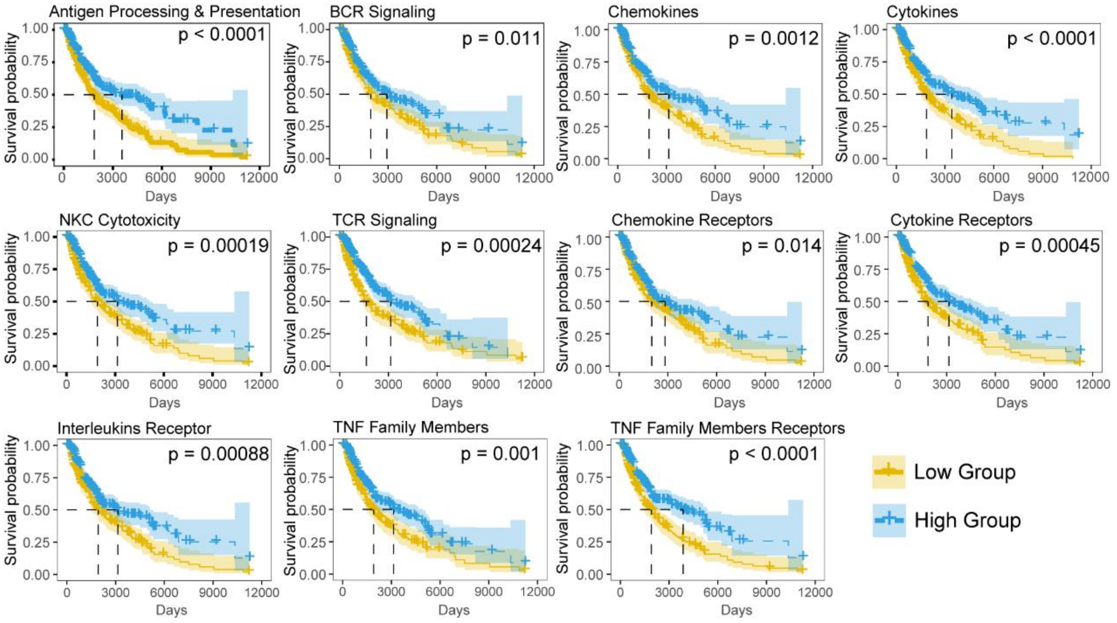

Supplementary Figure S4. KM curves of IRPs in SKCM.

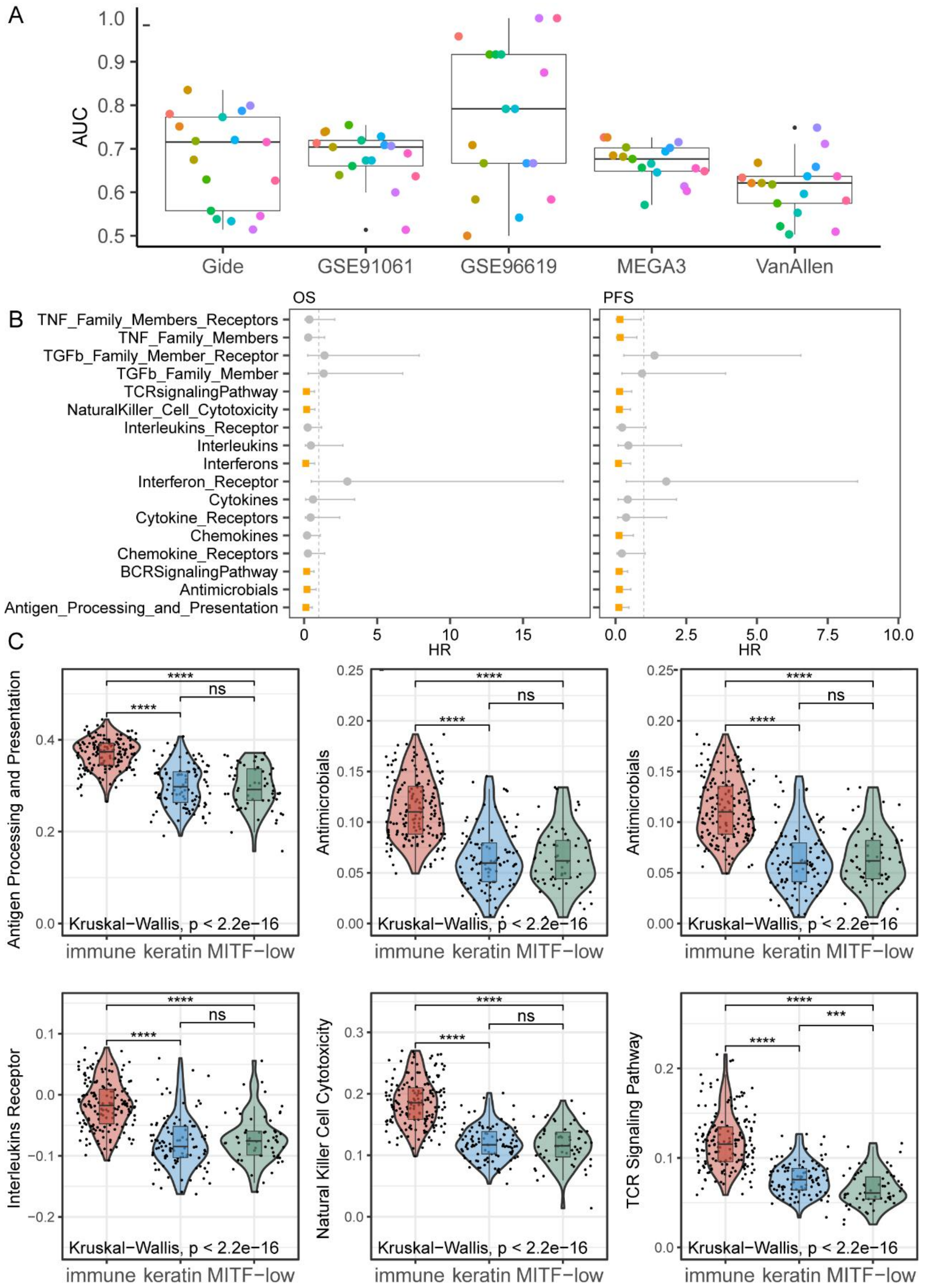

Supplementary Figure S5. A Boxplots of the area under the ROC curve (AUCs) in 5 cohorts. **B** Forest plot showing the results of Univariate Cox regression of OS and PFS in Gide cohort. Rectangle dots represent hazard ratio (HR) and the error bars show the 95% confidence intervals of the HR. Orange denotes statistically significant. **C** IRP scores among three SKCM phenotype.

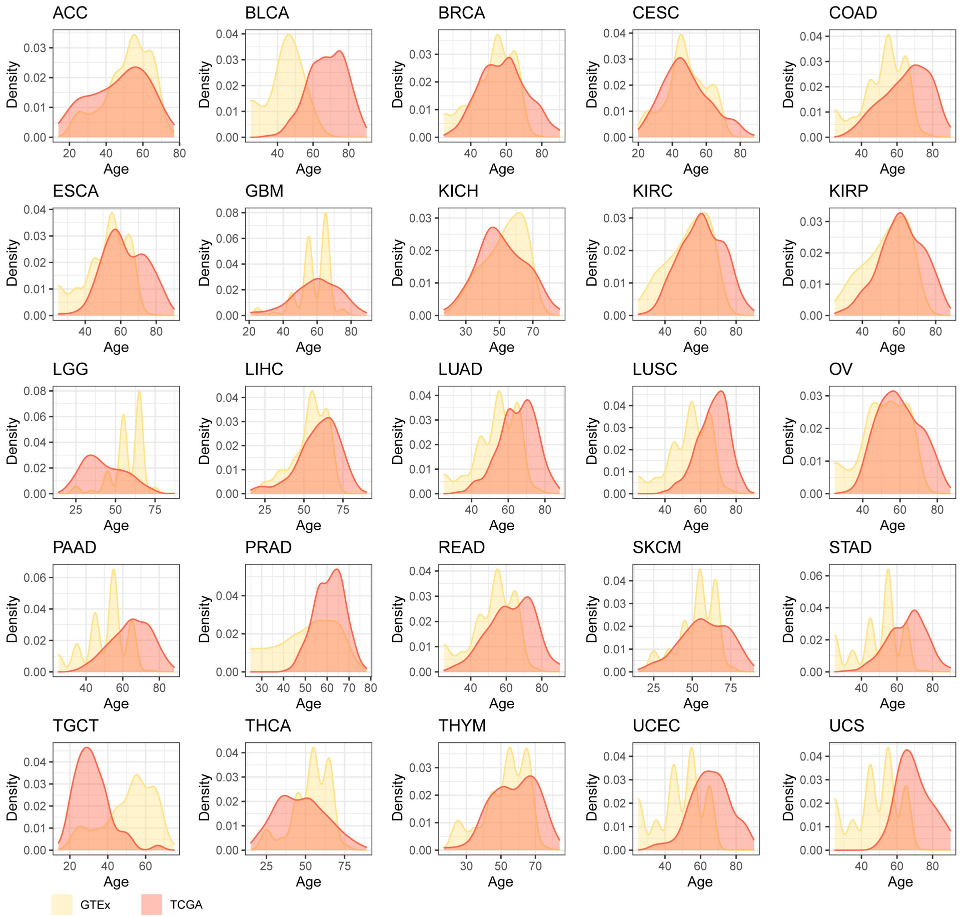

Supplementary Figure S6. Age distribution of samples from TCGA and GTEx samples.

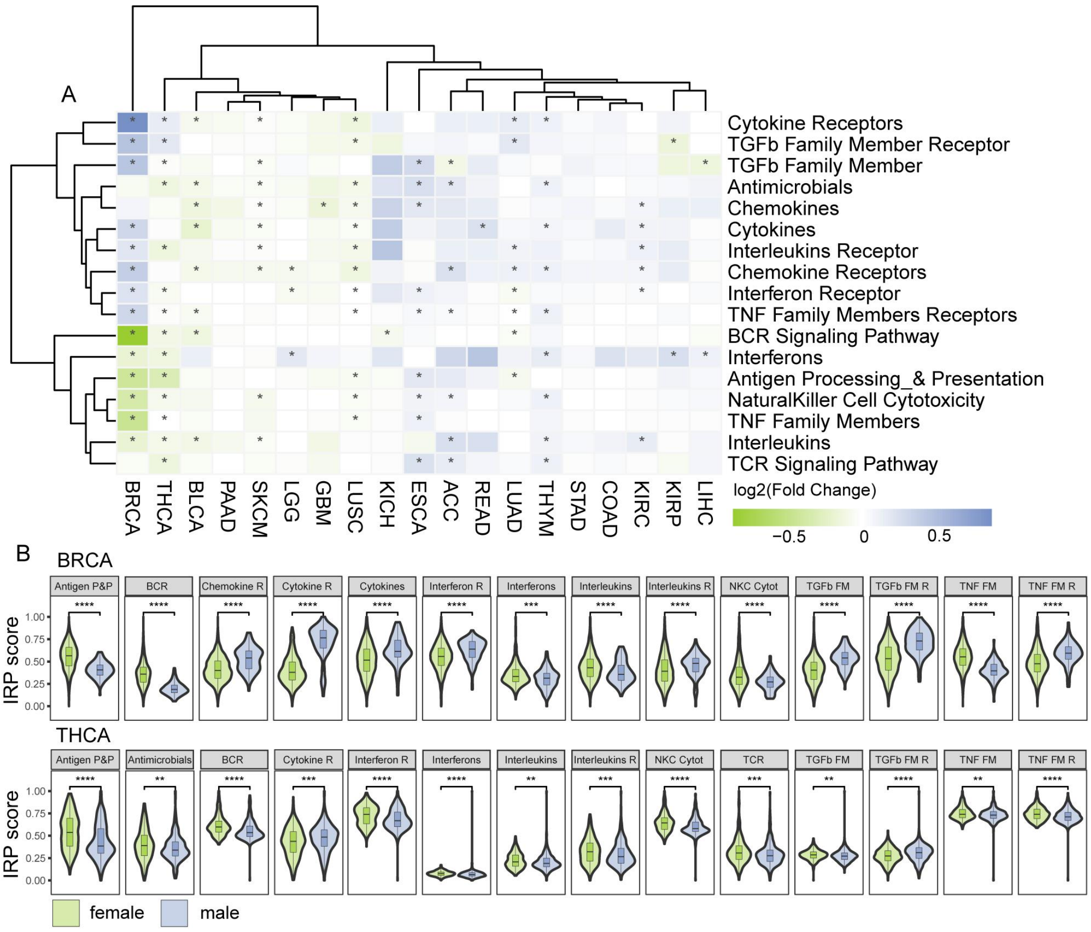

Supplementary Figure S7. Gender-related differences of IRPs. A Heatmap showing IRP expression level dysregulated in different gender groups across 19cancer types (Only cancer patients with more than one gender). '*' denotes a significantly dysregulation. B Boxplot showing the IRP differences between males and females in BRCA and THCA (wilcox.test $p < 0.05$).
